# Portal Annular Pancreas: Case Report of a Rare Anomaly

**DOI:** 10.7759/cureus.2366

**Published:** 2018-03-26

**Authors:** Baskaran Dhanapal, Gomathi Shankar, Balamourougan Krishnaraj, Ramkumar Govindarajalou, Jigish Ruparelia, Aniruthan Deivasigamani, Sarath Chandra Sistla

**Affiliations:** 1 Department of Surgery, Jawaharlal Institute of Postgraduate Medical Education and Research (JIPMER), Puducherry, IND; 2 Radiodiagnosis, Jawaharlal Institute of Postgraduate Medical Education and Research (JIPMER), Puducherry, IND

**Keywords:** circumportal pancreas, postoperative pancreatic fistula

## Abstract

Portal annular pancreas is a rare congenital anomaly in which the portal vein and/or the splenoportal confluence are completely encircled by aberrant pancreatic parenchyma. It is an asymptomatic condition and is usually an incidental finding. It is, however, important to a surgeon because the postoperative pancreatic fistula (POPF) rates following pancreatic resection are higher in patients with this anomaly.

A 47-year-old male presented with features of obstructive jaundice. He was diagnosed to have periampullary carcinoma, and pancreatoduodenectomy was planned. During surgery, uncinate process was seen extending posterior to the portal vein and was communicating with the body of pancreas to the left of the portal vein. After transection, there were two pancreatic stumps. The pancreatic duct was identified in the stump anterior to the portal vein. No duct was present in the posterior pancreatic stump. We closed the posterior pancreatic stump with interrupted polypropylene sutures and performed a duct to mucosa pancreaticojejunostomy in the anterior stump. On reviewing the preoperative computed tomography (CT) scan, we were able to identify the pancreatic tissue encasing the portal vein superior to the splenic vein.

Circumportal pancreas is classified based on the orientation of pancreatic duct to the portal vein and the relationship of the aberrant pancreatic tissue with the splenoportal confluence. Following pancreatoduodenectomy, the surgeon has to manage two pancreatic stumps, one anterior and the other posterior to the portal vein. No standardised technique has been described for management of the pancreatic stumps.

Every surgeon planning pancreatic surgery should be aware of this rare anomaly, and look for the same in the preoperative CT scan so that appropriate plan can be made regarding the type of pancreatic anastomosis.

## Introduction

Portal annular pancreas (circumportal pancreas) is a rare congenital anomaly of the pancreas in which the portal vein and/or the splenoportal confluence are completely encircled by aberrant pancreatic parenchyma. It has a prevalence of approximately 1.1 to 3.5%. It is an asymptomatic condition and is usually an incidental finding on abdominal imaging. But it is important to a surgeon because the postoperative pancreatic fistula (POPF) rates following pancreatic resection are higher in patients with circumportal pancreas than those with normal pancreas. Following pancreaticoduodenectomy, the surgeon has to manage two pancreatic stumps, one anterior and the other posterior to the portal vein. With the improvements in imaging modalities, this condition can be recognised in the preoperative contrast-enhanced computed tomography (CECT) scan. Every surgeon planning for pancreatic surgery should be aware of this anomaly and look for the same in the preoperative images. Here we present a case of periampullary carcinoma with portal annular pancreas who underwent pancreaticoduodenectomy.

## Case presentation

A 47-year-old male presented to us with features suggestive of obstructive jaundice for three months duration. He did not have cholangitis. Abdominal examination revealed hepatomegaly and a palpable gallbladder. The esophagogastroduodenoscopy revealed an ulcerated lesion in the ampullary region. Biopsy from the lesion had features of adenocarcinoma. His CECT scan showed a lesion in the ampullary region with dilatation of the common bile duct and pancreatic duct. There were no distant metastases. The portal and superior mesenteric veins and the superior mesenteric artery were free from the lesion. There was no abnormal arterial anatomy.

**Figure 1:**
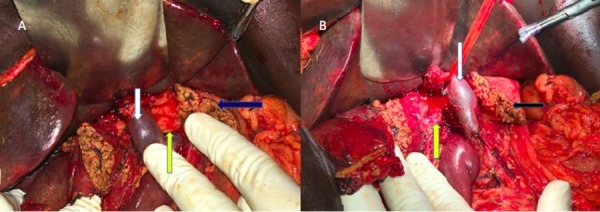
Operative finding after transection of the neck of pancreas demonstrating the aberrant pancreatic tissue (yellow arrow) posterior to portal vein (white arrow). The anterior pancreatic stump is labelled with blue arrow.

**Figure 2:**
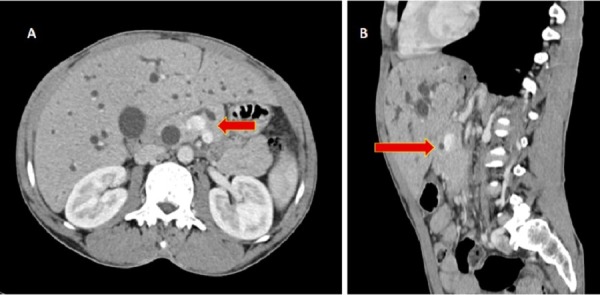
The coronal (A) and sagittal (B) views of the preoperative contrast enhanced computed tomography (CECT) scan demonstrating circumportal pancreas. The main pancreatic duct is anteportal.

Classical pancreatoduodenectomy (Whipple resection) was planned. During the uncinate dissection, we found that the uncinate process was extending posterior to the portal vein and was communicating with the body of pancreas to the left side of the portal vein (Figure 1A, 1B). We dissected the aberrant pancreatic tissue from the posterior surface of the portal vein, and then transected the pancreatic tissue anterior and posterior to the portal vein. After transection, we had two pancreatic stumps. The pancreatic duct was identified in the stump anterior to the portal vein. No duct was present in the posterior pancreatic stump. We performed "duct to mucosa" pancreatico-jejunostomy in the pancreatic stump anterior to the portal vein and closed the posterior pancreatic stump with interrupted polypropylene sutures. On reviewing the preoperative CECT scan, we were able to identify the pancreatic tissue encasing the portal vein superior to the splenic vein. The main pancreatic duct (MPD) was anterior to the portal vein. No duct was identified in the aberrant pancreatic parenchyma posterior to the portal vein (Figure 2A, 2B). He had secondary haemorrhage on postoperative day 7. On exploration, we found that there was bleeding from a tributary of the middle colic vein, which was secured. Subsequently, he had an uneventful recovery and he was started on adjuvant chemotherapy.

## Discussion

Portal annular pancreas is a rare congenital anomaly in which the portal vein and/or the splenoportal confluence are completely encased by the pancreatic parenchyma. The incidence of this anomaly is not known as there are no prospective studies. There are a few retrospective studies which have reported the prevalence of this anomaly to be around 1.1 to 3.5% [1,2].

The development of pancreas begins in the fourth week with the formation of dorsal and the ventral pancreatic buds. These are endodermal diverticulum arising from the primitive duodenum. The pancreatic parenchyma and the ducts arise from these buds. After the rotation of the duodenum to right, and formation of the “C” loop, the ventral bud occupies a position inferior and dorsal to the dorsal bud. The ventral bud forms the lower part of the head and the uncinate process. Rest of the gland develops from the dorsal bud. The fusion of the dorsal and the ventral pancreatic buds usually occurs to the right side of the developing portal vein. If the fusion takes place to the left side of the portal vein, then the uncinate process will extend dorsal to the portal vein and fuse with the dorsal part of the body of pancreas, resulting in a portal annular pancreas [3].

Circumportal pancreas is classified based on the orientation of pancreatic duct in relation to portal vein and the relationship of the aberrant pancreatic tissue with the splenoportal confluence. In type 1, the pancreatic duct is retroportal. Type 2 is circumportal pancreas associated with pancreas divisum. In type 3 the duct is anteportal. Each type is classified into subtypes based on the relationship of the aberrant pancreatic tissue with the splenoportal confluence as suprasplenic (subtype A), infrasplenic (subtype B), and mixed (subtype C). The most common type is type 3A [4].

The occurrence of hepatic arterial anomaly is similar to that in patients with normal pancreas. A CECT scan is adequate for diagnosing this condition [5]. But sometimes a thin rim of pancreatic tissue can be mistaken for tumor/node. Routine magnetic resonance cholangiopancreatography (MRCP) may not be needed if the ductal anatomy is clear in the CECT scan.

This is an asymptomatic anomaly. The clinical importance lies in the fact that POPF rate has been described to be around 44% to 46% in patients with circumportal pancreas [4]. There is no standardized technique described for the management of pancreatic stump in patients with circumportal pancreas. In a case with type 2A portal annular pancreas, the authors had ligated the posterior duct and performed a side to side pancreaticojejunostomy after transecting the neck of pancreas [4]. Some authors have described stapling of the posterior pancreatic stump and performing an anastomosis in the anterior pancreatic stump [4,5]. Some have described additional dissection and transection of pancreas to the left of portal vein so that the posterior pancreatic stump can be avoided [6].

## Conclusions

Every surgeon planning pancreatic surgery should be aware of this rare anomaly, and look for the same in the preoperative CECT scan so that the surgery team can be prepared for additional dissection around the portal vein, and appropriate plan can be made regarding the type of pancreatic anastomosis.
